# Medical Opinion and Movement

**Published:** 1910-07-16

**Authors:** 


### Medical Opinion and Movement.

### Noma.

An epidemic of noma came under the observation of Dr. Neuhof last year, and his conclusions about this now rare disease are published in the *American Journal of the Medical Sciences*. It is pointed out that formerly this disease was wont to break out in overcrowded and ill-regulated hospitals; nowadays it is much rarer, but has several times been reported from institutions which are excellent in every way. The pronounced association of noma with infectious fevers is well known, especially with measles and whooping-cough: in the present instance there were 81 cases of measles in the New York Hebrew Infant Asylum, and among these there developed eight cases of true noma and three doubtful ones. The disease, says Dr. Neuhof, is an entity, not a later stage of ulcerative stomatitis, though the latter favours its onset. There is regularly present a streptothrix characterised by a thick meshwork of mycelium at the border line between normal and necrotic tissue; fine rods and spirilla extend into the adjacent tissues. The constant presence of this streptothrix, to the exclusion of other organisms, indicates probably that it stands in direct ætiological relation to noma. The streptothrix is present before the disease is fully manifest; that is, in the pregangrenous stage, when alone radical treatment (by excision or actual cautery) should be practised. After the ulcer spreads the best results are obtained by conservative measures. General anæsthesia should never be employed because of the pronounced tendency to the development of septic pulmonary disease.

### The Treatment of Gonorrhœa.

The numerous methods by which the profession has endeavoured to shorten the course, alleviate the symptoms, and control the complications of gonorrhœa are exhaustively considered by Dr. Bangs in an address to the alumni of Bellevue Hospital, New York. His own experience leads him to the belief that irrigations with antiseptics do not readily control the symptoms, but are even liable to aggravate them. He is also convinced that they do not shorten or mitigate the attack, and that they favour posterior urethritis, infections of the prostate, vesicles, etc. They are inconvenient and sloppy; but for certain exceptional chronic cases he still admits their usefulness. With the albuminoid salts of silver Dr. Bangs is much better pleased. Especially if used in the early stages of the disease, they will in a large proportion of cases modify its progress and lessen the liability to complications. These salts are well under control; they can be used in the most intolerant urethra without aggravating the patient's condition, and they can safely be placed in the hands of the patient himself. The other important principle upon which he lays stress is that the patient himself must be treated: his habits, hygienic and social conditions, and general health must be supervised and rendered as favourable as possible. The patient's environment is just as important as the local treatment.

### Bromoform Poisoning.

In a very careful article published in the *Bristol Medico-Chirurgical Journal* Dr. Waterhouse analyses recorded cases of bromoform poisoning, including some under his own care, and pleads for the publication of all such instances as occur. It is chiefly for whooping-cough that this drug is ordered, and it is, in fact, esteemed greatly for this disease by several recognised authorities. The author commenced to use it for this purpose at the Royal United Hospital, Bath, in 1902, and was very satisfied with his results until in 1903 a child of four and a half years of age suffered severely from bromoform poisoning after taking the last dose in his bottle; the mother insisted that she had always been careful to shake the bottle as directed. In consequence of this the drug was abandoned for three years, until a severe epidemic began in which the failure of other remedies caused it to be again resorted to. An emulsion was accordingly made up by the hospital dispenser, using tincture of senega as recommended in Martindale's "Extra Pharmacopœia." This year another fairly severe case of bromoform poisoning has occurred, in a little girl taking slightly less than one minim of bromoform per dose. In this case it seems likely that the contemplated dose was not exceeded; nor is it probable that any decomposition of the mixture had taken place. The danger of the last dose of a drug so immiscible as bromoform has led on the Continent to the adoption of capsules as the only safe method of administration—an expedient well worthy of imitation in this country.

### Ovarian Epilepsy.

The connections between epilepsy and disease of the pelvic organs have been discussed in two of the Scottish journals lately: Mr. W. J. Rutherford deals with them in the *Glasgow Medical Journal* and Mr. H. S. Gibson in the *Edinburgh Medical Journal*. The latter confines himself to the results of ovariotomy in two cases of epilepsy which have come under his care. In one woman fits began six months after the onset of menstruation, and they occurred regularly with much dysmenorrhœa just preceding the flow, but never at any other time; they were controlled by 30 grains of bromide taken the night before menstruation was due. The left ovary was enlarged and tender, but pressure upon it did not evoke a fit. Removal of this ovary was followed by cessation of the epilepsy; one fit happened three months after the operation, but in the twelve months following there was no attack; the patient was 19 years of age. In the other case fits first appeared at the onset of menstruation; they recurred with the periods, but not every month, and never between them. Bromides controlled them. After curetting the epilepsy was in abeyance for a time, but eventually recurred worse than before; fits became frequent at all parts of the menstrual cycle, and bromides failed to control them. Double ovariotomy resulted in a cure which has been complete and permanent for three years so far. This author claims that in this menstrual type of epilepsy 66 per cent. can be cured, and 15 per cent. more improved, by operative treatment.

Mr. Rutherford's paper is concerned chiefly with the history of an extremely interesting case. This patient was a woman who began to menstruate at about 15 or 16 years of age. For three or four years previously she had had fits at fairly regular four-weekly intervals, but without any menstrual flow; the fits were typically epileptic. At the present time she has—unless on appropriate treatment—one fit about a week before the onset of menstruation, but for a year or two soon after puberty she had them exactly midway between the periods; this the author compares with what the Germans call *Mittelschmerz*. She has had four children, and during the amenorrhœa of pregnancy and lactation she has no fits, but when she weans her babies the fits reappear at once. Menstruation has always been regular and painless. The patient finds that by taking bromide for the week preceding the onset of menstruation the fits can be completely controlled, and she does not take it except during that time; she comes of a markedly epileptic stock. To Sir Halliday Croom's three forms of intermenstrual dysmenorrhœa the author would add a fourth class for these rare irregular cases characterised by nervous disturbance other than pain and without any uterine discharge. In view of the patient's personal and family history, it is perhaps open to argument that ovariotomy would have been good treatment, partly on the chance that it might cure the epilepsy, partly to prevent the procreation of epileptic progeny.

### The Patellar Reflex in Functional Disorders.

According to Wohlwill, in the *Neurologisches Centralblatt*, absence of the patellar reflexes in functional nervous disorders would seem to be generally admitted, though the condition is rare. But few cases have been reported, and in the majority of these the condition was not permanent, but showed variations in the presence of the reflexes from time to time. As an example, he reports a case of his own. The patient, a girl of twelve, had for two years before examination been subject to convulsions which had suddenly ceased. She then developed attacks of apprehensiveness accompanied by peculiar sensations as if she must fly or fall. At times the attacks were repeated frequently in the course of a single day. No other symptoms were complained of. On examination both patellar and Achilles reflexes were absent. A diminished tonus of the muscles of both legs was noticed. Pain sensibility was diminished generally. Pupil reflexes were normal, and in all other respects the results of neurological examination were negative, as were also lumbar puncture and Wassermann's reaction. On a second examination three days later patellar and Achilles reflexes were present and brisk, and muscle tonus was normal. During the following six weeks repeated examinations were made at frequent intervals, and on some occasions either the right or left patellar reflexes were found to be present, though never both at the same time, and twice it was noticed that both were absent. The Achilles reflex behaved in a similar manner. As the result of psycho-therapeutic treatment the attacks ceased, but the patellar reflexes continued to be absent. Treatment was discontinued after six weeks, and a month later both reflexes were normal. Seen again after another month, only the right reflex was present, and the attacks had again returned. Since he had excluded to his own satisfaction the presence of an organic disease, the author regards the condition as a functional one, though he is not sure that it is hysterical. He feels that any attempt to explain it on physiological grounds would rest on pure hypothesis, but points out that the case does not bear out Steiner's explanations, who maintains that the absence of these reflexes is associated with sensory anæsthesia.

### Non-absorbable v. Absorbable Sutures.

Madlener, in the *Centralblatt f. Chirurgie*, maintains that if a suture of sufficiently small calibre be used, better results are obtained with non-absorbable suture material, whether prepared by the aseptic or the antiseptic method, than with absorbable suture material. He prefers Rami thread to silk for all suture purposes, except in operations for uterine prolapse, vesico-vaginal fistula, and supra-pubic cystotomy. In these cases there is risk of the thread acting as a nucleus for the formation of a foreign body. He employs Rami fibre No. 0 for intestinal sutures, No. 1 for almost all ligature purposes, and No. 2 for ordinary stitches. In 757 operations performed during the last two and a half years he has exclusively used this material, and with the exception of two fatal cases, only one of his 124 hernia operations failed to heal by primary intention. In three out of sixty-eight goître operations suppuration occurred, which is a low average. He believes that the chemotactic effects of catgut serve to prolong secretion from the wound, and so to delay healing.

### Nail Extension in Fractures.

Professor W. Anschutz, in the *Deutsche Zeitschr. f. Chir.*, recommends that, in cases where ordinary methods of extension have proved unsatisfactory, a nail should be driven into the distal fragment, by means of which satisfactory extension can then be obtained. Enormous traction can be applied, and even in chronic cases the shortening will be rapidly overcome. He declares that the method is not painful, but it has the disadvantage that soft bones may be split by the nail. This can be obviated by driving the nail into the compact diaphysis at some distance from the epiphysis. He makes light of possible infection by the insertion of the nail through the skin. He has obtained excellent results in two cases of pseudarthrosis, the ends of the bone rapidly joining by callus-formation. In a case of shortening of the femur this was reduced from 9 cm. to 7 cm., although treatment was only instituted twenty weeks after the injury. He maintains that the method is a valuable resource when other methods have failed.

### Vaginal Douches of Lactic Acid.

As the result of numerous investigations it has been shown that lactic acid is present in the normal vagina up to a strength of 0.3 to 0.5 per thousand. Dr. N. Cukor, in the *Klin. Therap. Woch.*, argues from this fact that lactic acid should be used in vaginal douches in place of lysol, bichloride, and the rest of the ordinary drugs used, which are all more or less irritating. He believes that healthy women should use these douches daily to replace the lactic acid lost after bathing, etc., and declares that they hasten the return to the normal condition after menstruation. In cases of much discharge from the uterus or cervix their employment is indicated. Erosions of the cervix and catarrhal conditions of the genital apparatus are rapidly cured by them, and they can effectually be made use of in the place of all other antiseptic solutions.

### Eye Affections in the Insane.

In the *Crónica Médico-Quirurgica de la Habana* Dr. Fernandez describes the affections of the eyes he has met with in examining some two hundred insane patients. While diseases of the lachrymal apparatus are not very common, blepharitis is frequently seen, which he attributes to the habits of this kind of patient. Ectropion is exceedingly common, due, he thinks, to the frequency with which the insane strike each other. He met with few cases of trachoma despite the increase of the disease in Cuba of late. Pterygium was frequent, as were corneal affections. Cataract was common, and he operated on four cases with good results. He expresses his astonishment at the number of cases in which he came across increased retinal sensibility.

### Relation of Trauma to Cancer.

A very exhaustive discussion of the association of a local injury with subsequent development of a malignant growth (sarcoma is excluded from consideration) is contributed to the *Annals of Surgery* by Dr. Charles Phelps. On the whole, he sums up decidedly against those who trace a causal connection between trauma and carcinoma. Cancer, he says, is primarily dependent upon a cause which is congenital, is hereditary in a certain proportion of cases, and is as inexplicable as the force which determines *ab ovo* the future sex and peculiarities of the individual. Its development is favoured by various indeterminate and non-essential conditions; the proximal cause is as yet entirely unknown, and its future determination will depend upon the possible verification of a parasitic infection. The importance of the subject has been enhanced by recent medico-legal cases, wherein it has been sought to identify long antecedent trauma as the exciting cause of subsequent malignant change. But Dr. Phelps says that the more extended and carefully compiled statistics have been, the smaller the percentage of antecedent injury has been found; and that while contusions more or less severe are of constant occurrence in every part of the body, it is only exceptionally and when confined to the female breast that they have been supposed to produce carcinoma. Chronic inflammation is, however, quite a different matter; and there is reason to accept at their face value those cases in which trauma of whatever character when causing inflammatory exudation is apparently the indirect cause of malignant degeneration.

### Evidence of Respiration.

After carefully reviewing the literature relative to the Breslau test, Hobohm publishes the following conclusions in the *Vierteljahrsch f. Gericht. Med.* No matter whether the body be fresh or decomposing, distension of the stomach and first part of the duodenum may always be regarded as evidence of life. No medico-legal importance is to be attached to possible intra-uterine respiration, for in order that air may reach the intestine it is necessary that the child should not die immediately after birth. When intra-uterine respiration and artificial inflation can be definitely excluded the presence of air in the stomach alone strongly suggests live birth. It must, however, be remembered that a few bubbles of air in the stomach without distension and buoyancy of the organ when placed in water do not by themselves constitute evidence of respiration. The gases formed by putrefactive processes never give rise to a uniform distension of the lumen. Lastly, in cases where a positive result has been obtained from the lung test, absence of air in the stomach is of no value as negative evidence of respiration.

### Poisoning by Santonin.

Dr. Neer, of Vinita, Okla., reports in the *Critic and Guide* a case of fatal santonin poisoning in a girl two years old. The child had been given a teaspoonful of a "patent" vermifuge twice daily for three days. There was no indication of the amount of santonin in a given quantity of the fluid in the bottle, but the dose recommended was a teaspoonful for a child of two or over, and half that quantity for a younger child. A few hours after the last dose had been administered the child was seized with generalised convulsions, which continued at intervals until death. There was no fever, no evidence of digestive disturbance, and no meningitis. The child never regained consciousness. The pupils were dilated, there was some sweating, and between spasms the muscles were completely relaxed. The bowels were emptied by an enema and a purge was given. The convulsions were controlled for an hour or two by bromides, chloral and paregoric. Towards the last the pulse became very weak, the respiration stertorous, and the temperature slightly raised. It was impossible to make an examination of the urine before death, which occurred on the second day. The author remarks that such a case illustrates forcibly the danger of indiscriminate administration of patent vermifuge mixtures containing santonin for the treatment of worms which are often not present at all. Santonin should be given with caution to debilitated children, as a dose of 2 grains has been known to be fatal to a feeble child five years of age.

